# A study into shared reading groups, with specific relation to religious reading

**DOI:** 10.3389/fpsyg.2022.1025914

**Published:** 2022-11-25

**Authors:** Esther Valora Harsh

**Affiliations:** ^1^Independent Researcher, Liverpool, United Kingdom; ^2^Centre for Research into Reading, Literature and Society (CRILS), University of Liverpool, Liverpool, United Kingdom

**Keywords:** shared reading, religious feeling, translation in religion, Marilynne Robinson, literature and religion

## Abstract

This paper examines a live shared-reading group conducted through The Reader Organization, with the approval of the University of Liverpool's ethics committee. It is a revised excerpt from a successful inter-disciplinary Ph.D. thesis undertaken within the School of Psychology.^1^ The intention in forming the group was to explore the reading of Marilynne Robinson's *Home* by a wide variety of modern readers of different backgrounds and persuasions, in the light of religious writing in an age of diminished religious tradition. The main research question was to test what literature can do in carrying meaning which can be seen as religious, or was previously deemed religious, among readers who may not think of themselves in such terms. The second was to see how a shared community-group setting can enable collaborative engagement with the challenge to develop different ways of thinking, beyond the individual default of either religious dogma or anti-religious prejudice. The method employed overall in the wider Ph.D. study was Grounded Theory: essentially, empirical analysis rather than top-down conceptualization. Grounded Theory, in refusing to begin from rigidly preassembled categories, is appropriate to a literature-inflected study and, in particular, a literary study that is concerned with religious meaning in situations of humanitarian crisis. It allows the possibility of empirical work and careful detailed analysis, amid a complex of overlapping psychological, spiritual, and family concerns entangled within the experience of modern life. In this particular case study, which may be described as a form of Action Research, the researcher, also acting as the reader leader of the group, brought developed tools taken from psychologist Wilfred Bion, introduced to the reading group itself during the sessions as a means of measurement and navigation through the novel. If the aim was simply to undertake a study of the text, then this paper would be more narrowly literary, but the concern was with wider real-world effects in relation to individuals within the group work. Through close examination of the week-by-week transcripts of the reading group, this study highlights the search for moments of development, or what might have stood in the way of development. The researcher used a consensus group of three supervisors to check the selection of the best moments (failing or succeeding in coming closer to what will be called below, after Bion, “0”) recorded in the written transcripts of the sessions. One of the most powerful findings in this study is what will be called a mini-tradition developed by the group members in praxis, in terms of practices which they find, use, and come back to during their work with more difficult and painful passages in the text.

## The group-reading of *Home*

The following was research carried out during my Ph.D. study, combined with practical work with The Reader. Since the completion of the Ph.D., I now work as a practitioner at The Reader and a member of the Teaching and Learning Team. The research praxis undertaken with the shared reading group was conducted through The Reader Organization, where I took the place of the group leader, with the approval of the University of Liverpool's ethics committee. The Reader is an award-winning charitable social enterprise working to connect people with great literature and with each other. Its mission is to build a reading revolution and create environments where personal responses to books are freely shared in reading communities within many different outreach settings. Beginning life as a small outreach unit at the University of Liverpool in 1997, this national charity (established in 2008) pioneered the weekly “read aloud” model at the heart of its Get into Reading project, now also known as Shared Reading. The Reader currently has over 1,000 volunteers and partners, bringing over 2,500 people together each month to share and discuss great novels, plays, and poems in all four corners of the UK. Sessions take place in a variety of locations, including hospitals, prisons, corporate boardrooms, schools, GP surgeries, libraries, community centers, care homes, and supermarkets.

The stimulating, friendly, and non-pressured environments provide stability, support, and enjoyment for people who attend, establishing shared meaning and connections across social, educational, religious, and cultural boundaries. Previous evaluations have shown how The Reader's work is helping to improve wellbeing and reduce isolation, through using live literature as a vehicle for the search for meaning.^2^

Through both the writing and reading of literature, the finding of specifics, particular situations, strong emotions, and anomalies, both evoke and challenge a reader's customary frameworks and defaults. Here, that is what the act of reading is, a challenge that unfolds week after week. This study is also about weekly movements which are not simply progressive or straightforward, but erratic and part of the general unpredictability of the experiment.

In relation to this complexity, there are two (in my argument, necessarily) blurred territories in this action research. The first is the blurring between myself as a research analyst and leader/facilitator of the group, which made explicit to the group from the outset, for example, in discussing the use of Bion and the idea of “0” as first practiced in relation to psychoanalytic sessions. The second lies between the role of group facilitator (in the most neutral sense, simply, for example not allowing people to interrupt each other) and something more active as an enabler and guider. Again, I shared this with the group as part of the concerns in The Reader in general: to create a safe space but also to find legitimate means to help lead and encourage people to places of linguistic and psychological exploration beyond habitual norms or paraphrase. These two sets of concerns are held in tension, but that is part of the experiment involved in this case study and in need of further research.

I chose an area that is potentially volatile in relation to religion, where what I was most emphatically *not* interested in was the reinforcements of belief or non-belief: that is to say (as in any act of reading), people merely staying with their opinions and their defaults in a static manner. Nor had I any interest in replicating my own beliefs. My role was to ensure that the group remained concentrated on what they chose as key moments in the text: as safeguards (1), the use of Bion's “0” served as a means of pointing to key moments without recourse to a dogmatic or controversial vocabulary; while (2) the use of my consensus group of supervisors, in relation to transcripts, enabled scrutiny of the status of those moments and the possibility of unintended bias. This case history is offered even so as an experiment in risk and venture in a messily powerful area of human concern.

## Feeling “0”

I should say something more about why I used a navigational tool throughout this experiment from the psychology of Wilfred Bion.^3^ When vocabulary can be loaded with too many inherited implications, Bion wanted to try to use notations, letters, and algebraic indicators, instead of premature nouns and categories, to navigate feeling. As a psychoanalyst, he sought to give intensity a blind point, without giving it a name that begins to impose interpretation. This helps steer a humane agnostic pathway (religious or not) between a silenced first language and its possible recreation in a second form in common life: that is, it implicitly asks “Am I nearer or further away from the really real, when this happens or that is said?” Reading with Bion's dumb pointing tools within the experimental model of human existence called literature, and moving this way and that within its complexity, helps *find a language* for the densely mixed-up considerations and entangling circumstances within which the group must function. As with Bion and his patients, I think we can tell from the transcripts, as from the novel itself, when people are using certain elements of themselves that are routine or defensive, and when some other elements are coming in that are more spontaneous or unconscious and disruptive of defaults. I subsequently shared some of these thoughts about Bion with the group, in terms of providing a tool for pointing toward powerful places. Even though (as Bion says) the sense of total reality or truth can never be fully available to us, I told them, his “it” or “0” of the really real marks the significant moments in human beings—even if they're terrible, no matter: they are holding places for the primary secrets of existence in birth, family, marriage, crisis, aging, and dying. I was interested to see how the tool might help the group itself to be able to point initially to unbearable areas in a painfully intense novel rather than explain them secondarily. Hence, Bion was an agnostic guide in relation to a religious text, and as an indicator of moving nearer to or further from a moment of emergence of sudden new or powerful thought and feeling in the group. Some of the members of the group reported its usefulness at times, as a means of initial blind pointing, in place of having to find an explanatory language.

The shared reading group in this study consisted of seven women aged 45–85 years from around the Liverpool community, who responded to the advertisement of the group being formed as part of an experiment through the University and The Reader, and took place over 16 weeks.^4^ Of the seven women, as I discovered only indirectly and through the course of the actual discussion, four were to any degree what they would call “religious”. Four were familiar with the practice of shared reading, while the others experienced it for the first time. I offer below crucial moments and thematic concerns, from three separate shared reading sessions mainly in chronological order of development.

## Secondary motions

The continual initial challenge I encountered with the group was a recurrent inability to get anything primary or personal out of the text at points when we would pause from reading. The novel *Home* goes into emotional areas that often felt too hard to handle, and the participants at times explicitly indicating that these were places that were uncomfortable to speak about. Contrary to my initial hypothesis, it was not the religious element that seemed to be inhibiting my readers, but rather that the religious setting so far from comforting or curing the pain was allowing if not requiring its full force in the Broughton family. Where passages would come close to “0”, the group would move the discussion away from it. The group would most often default into speaking more about the characters, often externally and judgmentally, or offer a commentary *on* the story rather than feeling, thinking, and imagining *within it*.

Here is a particularly telling example from one of the painful passages of the novel. It comes near the end of the session at week 9 when largely the work was getting better: however, because it failed so badly in relation to a vital passage, it stands as a regression back to and a summary of what had been going not-so-well in the first month. In the novel *Home* set in a mid-twentieth century small town in Iowa, the old father and minister Boughton is at home being looked after by his kindly daughter Glory. Jack, the wandering lost child or black sheep of the family, has finally returned home after years of absence and profligacy, to visit the dying father who has despaired of him. The context is of a family, but a religious family.

### Week 9

The old man said “You take your time. But I want you to give me your hand now.” And he took Jack's hand and moved it gently toward himself, so he could study the face Jack would have hidden from him. “Yes,” he said, “here you are.” He laid the hand against his chest. “You feel that heart in there? My life became your life, like lighting one candle from another. Isn't that a mystery? I've thought about it many times. And yet you always did the opposite of what I hoped for, the exact opposite. So I tried not to hope for anything at all, except that we wouldn't lose you. So of course we did. That was the one hope I couldn't put aside” (*Home*, p. 121).

**Kate**: The father is apologizing and then the big turn against his son. It changed.

**Lily**: It is difficult because I almost feel for the father.

**Kate**: I did, before the *end*.

**Lily**: Yes that is true, and also the father is doing what he said Jack did. The opposite, the exact opposite of what was hoped for and needed. He cannot blame his son, he says, when he is still judging him so much that he cannot let anything go.

**Margaret**: But as the father says, he has known all of Jack's life that his son hasn't felt joy or happiness. And that would be hard to know that and carry that as the father.

**Elizabeth**: But if you really cared or loved your son, you would express concern, but you would do anything you possibly could to hold back your own feelings, or how it might have impacted yourself. He could have just left it there.

Lily starts in the right area with “difficult” and “almost” because she is recognizing that more than one feeling is happening, more than one family point of view or one easy direction being followed here with the father feeling real pain. However, it gets cut off by Kate, and then Lily joins her in commenting *on* the father, rather than trying to be *with* the father, or at least imagining what the father might be going through. Margaret makes a good attempt to get into the moment with the father, “that would be hard [i.e. painful] to know”, and in going on to a further deeper level of imagining in “hard to know and ***carry*** that as the father” – not only to know it but to have it, bear it, and feel it. The syntax and emphases show her getting closer to “0” here. But it is not to be sustained: the secondary idea of a parent holding back feelings itself holds Elizabeth back: “He could have just left it there”. But this book is never about “just leaving it there”. Nor is it about making blame for the father a way of avoiding the worst; it seeks the primary “0”.

The character of the father and even Glory are also frequently assessed through this secondary feeling (the supposed norm of “But if you really cared or loved your son...”) instead of the painfully real primary. The group itself will read about the movement from secondary to primary movements, and as a leader, I may point them out, but in their discussion, they will mainly stay within the secondary—which literature itself is meant to overcome. They will speak about the excusable complexities of being Jack with such a father, using a sort of humane compassion, but not more sympathetically imagining what it would be like to feel the damnation Jack is experiencing.

My initial conclusion at this first difficult stage of the group trying to “get into” the book was this: The pressure to seek recourse to the secondary is often naturally too strong, especially in the first month or so. When left to their own devices, the group will characteristically end up in that mode, especially when *Home* is felt as almost unbearable. The reader leader could remain a mere facilitator. But often The Reader urges a leader to step in and take part, to model a braver response and do more justice to the text; doing everything possible at least to point to the places and explore traces of the real, and not just their paraphraseable aftermath; to point to the inside and not just the external. In a wider sense, getting out of the secondary mode is the first thing that has to be done emotionally in reading; nothing of value can take place otherwise.

Crucially, this novel in particular is not designed to be satisfied with commentary, explaining away every human suffering. It cannot seem to settle for any understanding achieved by retreat or by means of a safety barrier between reader and text.

## Form: “Double listening”

One of the significant transformations is when a reader is not just commenting upon what is *in* the text, but working out a thought that springs from the text and is bigger than its immediate occasion.

In week 6, the group has just read how Jack has been helping Glory in the garden all day. He got a splinter from using the gardening tool, and the reverend made a big fuss to make sure he helped Jack with the small wound. Now they are sitting at dinner where Father Boughton is carefully avoiding any possible questions that could be uncomfortable for Jack. Glory watches the situation, the avoidance of “0”, and the attempted use of secondary politeness's within the text itself:

### Week 6

Through supper Jack was patiently restless, hearing out his father's attempts at conversation.... Jack watched him with the expression of mild impassivity he wore now that the embarrassments of his arrival were more or less behind him. She felt sorry for her father, happy as he was. It was hard work talking to Jack. So little in his childhood and youth could be mentioned without discomfort, his 20-year silence was his to speak about if he chose to, but they were prepared to appreciate his discretion if any account of it might have caused more discomfort still. Then, there was the question “Why are you here?” which they would never ask. Glory thought, Why am I here? How cruel it would be to ask me that (*Home*, pp. 65–6).

**Jackie**: So uncomfortable. Why are they always so uncomfortable around one another?

**Group Leader**: Yes, very uncomfortable. Which parts do you think are the most uncomfortable?

**Jackie**: You wonder if they will ever be at ease with each other. Before dinner the father acts as if he is really worried when Jack gets a splinter, he wants to be the one to help it, but it feels awkward…his concern feels awkward. He has been trying Jack's whole life to build bridges, and it's never natural! Why do they keep *trying*? It feels so uncomfortable the more they try. It feels like underneath all these attempts it just always makes things more uncomfortable for everybody.

**Group Leader**: I think this unspoken underneath is important. What area of the text did you feel it the most?

**Jackie**: It's just that on top of this feeling they seem to be only acting out the parts of a family relationship, you know? Only on top. “So little in his childhood and youth could be mentioned without *discomfort*” then later “*more discomfort still*”

**Audrey**: Hmm, more discomfort …but I think Jack is more *sincere* in his trying! He may feel uncomfortable, but he is also showing the respect by listening. And earlier that day it said that he ‘rolled up his sleeves' and helped with the gardening. I think he is listening sincerely, even if it is uncomfortable.

**Kate**: Ah, I know, it looks like Jack is just surviving the moment.

**Group Leader**: Hmm yes, “restless”.

**Kate**: Yes, and “mild impassivity” But I wonder if there is more going on underneath Jack that we just can't get to…or we just can't know about. [She pauses] See here: “Jack was patiently restless, *hearing out* his father's attempt at conversation” I wonder if Jack is not only trying to get through it, but actually underneath it all, I wonder if he might be listening to them…I mean listening maybe about what it would *be like* for them—Glory and especially the reverend—to see him and talk to him. Maybe he knows he is bringing back a difficult situation in himself.

**Group Leader**: Wow, that is interesting Kate, to be able to imagine how the people around you are listening to you, and what it is like for them on the receiving end.

**Michelle**: That's like double listening.

**Group Leader**: Yes! Like more than one thought happening at once, in different directions too.

**Kate**: It is, and I don't know how to always exist in that, or if that is what's happening here, but I wonder what that would be like for Jack if it was happening.

The group is now making something together, adding layers to each other's sentences, and getting momentum from each other's thoughts. Certain group members fall into instinctively performing certain functions: Jackie's questioning, Audrey looking to pull out anything sincere in the midst of awkwardness, Kate doing the digging in, the working out of something implicit, and Michelle bringing everything together to try to seal the exciting thought. Those functions are not permanent: though temperamentally or intellectually one person may be more suited to one particular function than another, the functions can move around from person to person in the light of a particular context and occasion. It is, at any rate, the most imaginative move in developing thought that has come about in this group. They begin to imagine not only what is *not* said but, *via* Kate, what it is like to imagine how others have to deal with one's presence and silence. Michelle's powerful “That's like double listening” clinches it. For Jack is both the subject and object here. Jack #1 as the subject has his own feelings, but as Jack #2, especially on his return home, he imagines the others' feelings about him as (so to speak, grammatical) object, and then has to take 2 back into 1, subject and object at once, with a rebounding effect on his own feelings, as Jack #3. He listens to them in pain, and in more pain, he imagines how they listen to him and what they hear inside their own heads in response. In that position, he has to bear that double consciousness of being a creature in the world who is both an “I” and a “you”,^5^ being alone and consciously feeling that loneliness, even amidst others, with the added guilt of a new realization of his long-continued effect upon them. It is a terrible complex overload to “carry”, to use a favored term of the group members.

And this twist and turn of shape, this shift of centers, applies to the novelist as well as to her character, as she uses something like human geometry to mark the turns: “I think of Fiction of having dimensionality: you don't make a simple statement, you rotate an idea and look at it from various sides.”^6^ Double listening for Jack is like that rotation of ideas, another instance of form taking the place of simple narrative, of linear straightforwardness. If one point of view is a formal place from which to start, then double listening is that form altering in the midst of itself. The moment the form has changed and densened in that way, the novel is closer through Jack to imagining “0”, listening to what George Eliot called “the roar on the other side of silence”.^7^

## Backward to primary

One of the most exciting discoveries in this project came about just when the group seemed blocked toward primary feeling. We have already seen in week 9 an especially moving yet painful passage managed by the readers' default of blaming Jack's father. Time was short and we did not have the opportunity at that point in the session to revisit and dig deeper, so in week 10, instead of moving forward despite the disappointment, I chose to go back and try again to find another way forward to a feeling that would reach the center of the pain felt in *Home*. Going forward linearly would have felt like going away from and completely ignoring the failed feeling; turning backward felt like the only hope to move forward:

### Week 10

From the group leader's weekly write-up diary:

*As the group members came in, each mentioned something about last week's reading. Since the group didn't have a lot of time to get into the passage the week before, I thought it would be important to go back to it*.

**Group Leader**: Before we start this next section this week, I wanted to ask if there has been any more thinking from last week? I know we ended on that really painful moment with Jack and the father. We didn't have very much time to get into it. Any more feelings from it?

(silence for about 20 seconds)

**Kate**: I was thinking about how Jack laughs earlier in the passage. He laughs. Why does he laugh? I've been wondering.

**Michelle**: It's like a nervous laugh he has isn't it? He doesn't mean to laugh, but he does.

**Group Leader**: Yes, why does he laugh?

**Michelle**: It's like when…when something awful happens you just…

**Audrey**: He puts his hand over his face.

**Lily**: Yes, throughout it keeps saying “and Jack laughed”, and it is usually during very serious times. But I don't think he is genuinely laughing, do you?

**Kate**: It's just a way of deflecting it, don't you think?

**Group Leader**: Ah, deflecting it. Deflecting it…what is it that he is deflecting, do you think?

**Kate**: Well it's…it's becomes too much for him.

**Audrey**: Can we read that bit again?

“And why am I talking to you about this? But it was always a mystery to me. Be strict! People would say that to me. Lay down the law! Do it for his sake! But I always felt it was a sadness I was dealing with, a sort of heavyheartedness. In a child! And how could I be angry at that? I should have known how to help you with it.”

“You helped me. I mean, there are worse lives than mine. Mine could be worse.” He laughed and put his hand to his face.

“Oh yes. I'm sure of that, Jack. I see how kind you are now. Very polite. I notice that.”

“These last years I've been all right. Almost 10 years.”

“Well, that is wonderful. Now, do you forgive me for speaking to you this way?”

“Yes, sir. Of course I do. I will. If you give me a little time.”

The old man said “You take your time. But I want you to give me your hand now.” And he took Jack's hand and moved it gently toward himself, so he could study the face Jack would have hidden from him. “Yes,” he said, “here you are.” He laid the hand against his chest. ‘You feel that heart in there? My life became your life, like lighting one candle from another. Isn't that a mystery? I've thought about it many times. And yet you always did the opposite of what I hoped for, the exact opposite. So I tried not to hope for anything at all, except that we wouldn't lose you. So of course we did. That was the one hope I couldn't put aside.'

Jack withdrew his hand from his father's and put it to his face again. “This is very difficult,” he said. “What can I do–I mean, is there something I can do now?” (*Home*, pp.120-1).

**Michelle**: You know, something else from last week…I was thinking about the father actually. I think, I think the father is really being sincere. At the end there, the father was just baring his own soul. I don't think he is wanting to harm Jack with his words.

**Audrey**: Well I took it home and re-read it as you know, and it sounded to me exactly like that. You know, he was apologizing to his son for not giving him what he probably needed, or not investing in what he needed. In reading again, I think there is a different way to look at the father and what he is feeling in this moment.

**Lily**: Yes, I think I am usually pretty hard on the father because I cannot believe how he is sometimes, *but* that last part of the paragraph there, I have a hard time working it out.

**Group Leader**: That is interesting Lily. Yes I think it would be the easier thing to do to just say Jack is somehow good and the reverend is actually bad, but that doesn't seem to get to the right feeling here. As you say Michelle, the father is being very sincere in what he shares. It feels like he knows it might hurt (“you'll have to forgive me for this, Jack”), but he knows he needs to say it! It's been 20 years. I also think what you've said Lily is really interesting too about the last part of the paragraph. Shall we look at it again?

“So I tried not to hope for anything at all, except that we wouldn't lose you. So of course we did. That was the one hope I couldn't put aside.”

**Michelle**: Yes, I don't think you can really give up on the father from this.

**Lily**: But I just can't get around this! The last three parts: “except that we wouldn't lose you. So of course we did. That was the one hope I couldn't put aside”: I really struggle with it. It doesn't make sense to me, I feel like it is contradicting.

**Group Leader**: Yes, trying to count the thoughts, the three clauses, is another good way of trying to follow the thinking.

**Margaret**: He's saying he can accept anything from him, “but don't leave”. He has been carrying grief with hope all along. And the more hope he has had, the more grief comes back to him. But he can't stop having hope for his son. It's really sad. The father is trapped. The father is trying to tell the son that he is trapped because of his love for him.

**Kate**: You almost want to take out “so of course we did” so that it would read “except that we wouldn't lose you. That was the one hope I couldn't put aside” It looks less complex that way.

**Group Leader**: Ah, that would feel more straightforward, wouldn't it? What do you think that middle bit means—“so of course we did”?

**Elizabeth**: Well it is the most hurtful thing of all that they lost Jack, isn't it. And so if he set aside everything, except that hope…it would almost be like “so of course it would be that one thing that would be taken from me, wouldn't it?” It's a bit cynical. I hear men say this sometimes, but really there is pain behind it. Yes...

**Audrey**: Yes, I think there is a lot of pain behind these statements.

**Margaret**: And at the end there, you need to understand, it might have taken a lot out of him. To be able to say he is sorry, and he would've forgiven his son for anything, so why leave? Why leave? He would have forgiven him for anything! Like “you could have done anything, but I would have still wanted you to stay”. That's why he turns away from Jack. He is tired and embarrassed I think.

**Group Leader**: Ah, thanks for that Elizabeth and Margaret. I think it really does change things to step into what it might be like to feel these hard and painful things as Father Boughton. And that last bit of the paragraph is really something to try to work out. It is interesting that it is placed right there in the middle, as if we have to *go though it* in order to get to the end of the sentence. I think the word “except” sets it up to make “of course” and “couldn't put aside” even more painful to have to get through.

**Michelle**: Yes, I go to think one thing, and then another thing, and then even another big thing again. That's how I feel when we read this story. Sometimes the sentences in each paragraph just keep adding one thing on top of another, until it almost feels too much.

**Audrey**: Yes.

**Group Leader**: Yes! Too much! More than you can carry. I think in this moment the father is someone I cannot have ill feelings toward, because I feel too much of his own pain. We've spoken about the father almost passing on this grief and pain to his son, but as we read it, it feels like it is passing on to us as well! It can feel unbearable.

**Lily**: That is exactly it. *Unbearable*. But it is hard enough to carry what Jack is feeling.

**Kate**: Yes, that's it, a complete loss of words, or not knowing how to carry it all himself:

‘Jack withdrew his hand from his father's and put it to his face again. “This is very difficult,” he said. “What can I do–I mean, is there something I can do now?”

**Michelle**: It's like, before we could feel more what Jack is feeling, but now we are feeling more what the father is really feeling.

**Group Leader**: Yes, but to be the person that all these unbearable feeling are attached to, on top of the heavy reality you already feel on your own.

**Audrey**: And those words from Jack. Ooh it's like he still wants to help or make amends. What in his father's religion might be called repentance, forgiveness, even peace and grace. Needed from somewhere, somehow.

**Group Leader**: Yes, instead of going away, Jack is wanting to do something, or anything: “is there something I can do now?” to try to make it better, or to take this pain away that he's caused. But we are at the limits of what can be done to repair things.

**Michelle**: This is what I mean. It is all too much in different ways. For both of them at the same time.

(she laughs)

It is more than you usually handle in one story, isn't it?

Now that we had come back to the text closer to 0, it was important to hold this open as long as possible for anything to break through. The most important moment comes in Michelle's discovery: “Sometimes the sentences in each paragraph just keep adding one thing on top of another until it almost *feels too much*.” That is the closest the group has ever come to “0”, especially in terms of the use of sentences. It is a moment of real reading, going with the currents of “too much” even through the sentence syntax. Then, Lily speaks in a tone closer to “0” in “That is exactly it. *Unbearable”*. There are no longer simply separate people, or single thoughts or separate feelings, from the moment Lilly spoke of the father “carrying grief with hope all along”, and then counting the way three thoughts combined and morphed.

This feeling of passing on but carrying the weight of what is passed feels like the last thing anyone wants in the story. It is as if the novel and group must express the pain of not only carrying the pain but also the other pain of Jack's question “What can I do—I mean, is there something I can do now?” This is about trying to convert or translate the weight into action. It is a point where, in a religious novel, the help that is something like grace feels most needed, and it is nowhere to be found in and around this passage: this marks the exact point where it needs to be, but the father's pain cannot give it, Glory is not in the position to give it, and the readers in the group have to bear both pains, father's and son's, through the daughter's.

This, I conclude, is about transmission, but a painful kind of transmission as compared with the laying on of hands in a family. “My life became your life” would be the form of primal transmission. But what is passed on here is a more fallen tradition of family heartache and unresolved troubles, with layers and echoes attached. Within that, even so, there is the feeling of what is needed but missing.

Later, I circulated to the group, in the consolidation of their own efforts, what Marilynne Robinson herself wrote outside her novels:

From the human point of view, I think that when you participate in grace, you're elevated above worldly considerations— grudges, fears, resentments—all those things that you accumulate in the clutter of self-protectiveness that arises as you develop in life. The moments of grace are the moments in which your vision of reality is, for the moment, actually free. You are out of the trenches. And I think that is something that people very often feel they have experienced, that experientially it is true. I often talk to people who have no theological vocabulary, but the minute the concept of grace becomes available to them, they recognize it. They love it. It could so easily be the core of any sort of reconstruction of our religious sensibilities.^8^

For Robinson, what matters is a need that religion responds to because human psychology both requires and recognizes it. As with William James in *The Varieties of Religious Experience*, to which she considers herself indebted, it is less important to her whether religion is seen as a form of human psychology or psychology as an approach to lost religion: the meeting point lies in the living feeling of the human dilemma.

## Conclusion on reading methods

This comes out of the way the group regressed into fixed attitudes at the end of session 9 but refound their way by the middle of session 10: an epitome of what is at stake across the sessions as a whole. The best moments in the group feel like the nucleus for reviving and developing a reading tradition, in the past often associated with biblical exegesis, but here a sort of mini-tradition of deploying reading tools developed in the sessions themselves by this group. Tools are being spontaneously found here that will be needed in the future to navigate through secondary responses:

- **Getting away from defaults:** A movement from simple defaults and assumptions to get into real reading – specifics that may not conform to what participants may have previously wanted but whose force, when attended to, takes readers into a new situation. This is related to Marilynne Robinson's (theologically inflected) sense of the revelatory newness of occasions.
- **Pointing to places that matter emotionally:** This included a sense of when things were lovely as well as painful. Pointing is about instinctively locating specific places, as a primary action before any secondary articulation or explanation.
- **Feeling “0”:** This goes with pointing as a form of mute orientation. Without having formal language and without trying to avoid an encounter, one can just point to “it”, the place of most reality. In *Home*, it may be a place that is terrible, but also accepted as somewhere worth going to, often through following a difficult syntax. It can mark a development from “this is too painful” to something more like “the truth, at all costs”.
- **Form in place of a story or single character analysis:** This involves thinking of *more* than one thing at a time, of more than one character or one scene at a time but relationships. It is related to connecting backward and seeing how the novel is getting made again in the act of reading it.
- **Connecting:** About having more than one thought or point of view, and making links between two things (places, persons, ideas). This is most powerful when the links are made backward, in sudden excited retrospect. It is a higher development of pointing which is to do with the mobility of mind, and the capacity to remake the thinking of the novel by recreative memory.
- **Group becoming one mind:** The group members begin to form a sort of relay between each other, handing on thoughts to take them further. The group is working and picking up on each other's points, almost as though one cooperative mind. Just as the characters are not separate in the novel, so the members are not separate in the group.
- **Memory claims a creative role here:** Turning back in week 10 rather than going on sequentially: at the beginning of the session, readers remembered and reclaimed what had been too quickly or automatically in the previous week. Memory then looks to be more forward-pointing than backward, as it goes back to make a forward motion in search of a future for itself. Instead of losing their way, the group and the group leader tried to get closer to 0 again, feeling its loss, through which a renewed sense of development can find meaning and a future for itself.

All of these tools were used and re-used over the course of the shared reading group experience, becoming trusted practice. But some of them the group leader would need to bring back into the group, reminding the readers of their being useful ideas that had arisen out of practice and, converted into tools, could further inform it (e.g., “double listening” and “linking backwards”). It is important that good moments of praxis are not just left in time, as one-offs, but become mini-traditions of the practice of shared reading, consolidating confidence, and aiding creative development. I am interested to hold open the possibility of the group being able to recreate a means of attention that, as with Marilynne Robinson's own novel-work, salvages meaning from the breaking of religious tradition in the home of this novel, the novel and the group working together.

## Tradition renewed through shared reading

After the shared reading group experiment, I interviewed the founder of The Reader, Jane Davis, on shared reading and her own experience of reading *Home* which she considers her book of the century. Jane also hosted Marilynne Robinson at The Reader Organization's headquarters in Liverpool in 2011 where she did an informal session on *Home*. I showed her a draft of the findings reported earlier, to test them against her reaction. I asked Jane to appraise the concluding idea of a mini-tradition of reading and renewed through the shared reading groups. She agreed on these grounds:

1. The group leader is the passer-on of the readerly tradition, partly through “doing it”, modeling the act, but also recognizing and encouraging its emergence.
2. The aim is that, ideally, everyone in the group should become a reader in some deep traditional version of that term, as a seeker for meaning through its signs, seeing the spirit through the letters.
3. But between 1 and 2, it is not possible simply to pass on the tradition of being a reader: it has to be rediscovered and reinvented in living and spontaneous practice by a group carrying out live collaborative work, without guarantees of its success or lastingness.

The deepest readerly traditions have been established in relation to religious texts, such as the Bible. Here, Jane Davis argued, such attentive seriousness is redeployed in relation to non-sacred texts that take concerns that might have been deemed religious into areas of personal psychology and familiar relations. In the realm of psychology, stimulated by works as powerful as *Home*, readers whether religious, formally religious, or consciously non-religious do group work together in a shared feeling of meaning.

## Data availability statement

The original contributions presented in the study are included in the article/supplementary material, further inquiries can be directed to the corresponding author.

## Ethics statement

The studies involving human participants were reviewed and approved by University of Liverpool. The patients/participants provided their written informed consent to participate in this study.

## Author contributions

The author confirms being the sole contributor of this work and has approved it for publication.

## Funding

This research was self-funded as part of doctoral studies at the University of Liverpool.

## Conflict of interest

The author declares that the research was conducted in the absence of any commercial or financial relationships that could be construed as a potential conflict of interest.

## Publisher's note

All claims expressed in this article are solely those of the authors and do not necessarily represent those of their affiliated organizations, or those of the publisher, the editors and the reviewers. Any product that may be evaluated in this article, or claim that may be made by its manufacturer, is not guaranteed or endorsed by the publisher.

## Footnotes

2 The Reader, 2022.

3 Bion (1970). See also Bion (1965).

4 Participants all signed consent forms and were informed that they had the right to cease their participation in the experiment at any time. The names of all participants have been anonymized.

5 Buber, 1958.

6 Hope University, 2015.

7 Eliot, 1871.

8 Robinson (2016); hereafter cited as *Grace in Shakespeare*.
